# Clinical study of thoracoscopic assisted different surgical approaches for early thymoma: a meta-analysis

**DOI:** 10.1186/s12885-024-11832-7

**Published:** 2024-01-17

**Authors:** Jincheng Wang, Ti Tong, Kun Zhang, Haiping Guo, Yang Liu, Jindong Li, Haiyang Zhang, Quanqing Li, Zhenxiao Zhang, Yinghao Zhao

**Affiliations:** 1 Department of Thoracic Surgery, the Second Hospital of Jilin University, Jilin, China; 2 Jilin Provincial Key Laboratory on Molecular and Chemical Genetics, Kun Zhang, The Second Hospital of Jilin University, 130041 Changchun, Jilin China; 3 Department of Cardiac Surgery, the First Affiliated Hospital of Harbin Medical University, Harbin, China

**Keywords:** Early thymoma, Subxiphoid thoracoscopic thymectomy, Intercostal approach video thoracoscopic surgery, Modified subxiphoid thoracoscopic thymectomy

## Abstract

**Objective:**

The efficacy and safety of subxiphoid thoracoscopic thymectomy (SVATS) for early thymoma are unknown. The purposes of this meta-analysis were to evaluate the effectiveness and safety of SVATS for early thymoma, to compare it with unilateral intercostal approach video thoracoscopic surgery (IVATS) thymectomy, and to investigate the clinical efficacy of modified subxiphoid thoracoscopic thymectomy (MSVATS) for early anterior mediastinal thymoma.

**Methods:**

Original articles describing subxiphoid and unilateral intercostal approaches for thoracoscopic thymectomy to treat early thymoma published up to March 2023 were searched from PubMed, Embase, and the Cochrane Library. Standardized mean differences (SMDs) and 95% confidence intervals (CIs) were calculated and analyzed for heterogeneity. Clinical data were retrospectively collected from all Masaoka stage I and II thymoma patients who underwent modified subxiphoid and unilateral intercostal approach thoracoscopic thymectomies between September 2020 and March 2023. The operative time, intraoperative bleeding, postoperative drainage, extubation time, postoperative hospital stay, postoperative visual analog pain score (VAS), and postoperative complications were compared, and the clinical advantages of the modified subxiphoid approach for early-stage anterior mediastinal thymoma were analyzed.

**Results:**

A total of 1607 cases were included in the seven studies in this paper. Of these, 591 cases underwent SVATS thymectomies, and 1016 cases underwent IVATS thymectomies. SVATS thymectomy was compared with IVATS thymectomy in terms of age (SMD = − 0.09, 95% CI: −0.20 to − 0.03, I^2^ = 20%, *p* = 0.13), body mass index (BMI; SMD = − 0.10, 95% CI: −0.21 to − 0.01, I^2^ = 0%, *p* = 0.08), thymoma size (SMD = − 0.01, 95% CI: −0.01, I^2^ = 0%, *p* = 0.08), operative time (SMD = − 0.70, 95% CI: −1.43–0.03, I^2^ = 97%, *p* = 0.06), intraoperative bleeding (SMD = − 0.30. 95% CI: −0.66–0.06, I^2^ = 89%, *p* = 0.10), time to extubation (SMD = − 0.34, 95%CI: −0.73–0.05, I^2^ = 91%, *p* = 0.09), postoperative hospital stay (SMD = − 0.40, 95% CI: −0.93–0.12, I^2^ = 93%, *p* = 0.13), and postoperative complications (odds ratio [OR] = 0.94, 95% CI: 0.42–2.12, I^2^ = 57%, *p* = 0.88), which were not statistically significantly different between the SVATS and IVATS groups. However, the postoperative drainage in the SVATS group was less than that in the IVATS group (SMD = − 0.43, 95%CI: −0.84 to − 0.02, I^2^ = 88%, *p* = 0.04), and the difference was statistically significant. More importantly, the postoperative VAS was lower in the SVATS group on days 1 (SMD = − 1.73, 95%CI: −2.27 to − 1.19, I^2^ = 93%, *p* < 0.00001), 3 (SMD = − 1.88, 95%CI: −2.84 to − 0.81, I^2^ = 97%, *p* = 0.0005), and 7 (SMD = − 1.18, 95%CI: −2.28 to − 0.08, I^2^ = 97%, *p* = 0.04) than in the IVATS group, and these differences were statistically significant. A total of 117 patients undergoing thoracoscopic thymectomy for early thymoma in the Department of Thoracic Surgery of the Second Hospital of Jilin University were retrospectively collected and included in the analysis, for which a modified subxiphoid approach was used in 42 cases and a unilateral intercostal approach was used in 75 cases. The differences between the two groups (MSVATS vs. IVATS) in general clinical characteristics such as age, sex, tumor diameter, Masaoka stage, Word Health Organization (WHO) stage, and intraoperative and postoperative conditions, including operative time, postoperative drainage, extubation time, postoperative hospital stay, and postoperative complication rates, were not statistically significant (*p* > 0.05), while BMI, intraoperative bleeding, and VAS on postoperative days 1, 3, and 7 were all statistically significant (*p* < 0.05) in the MSVATS group compared with the IVATS group.

**Conclusion:**

The meta-analysis showed that the conventional subxiphoid approach was superior in terms of postoperative drainage and postoperative VAS pain scores compared with the unilateral intercostal approach. Moreover, the modified subxiphoid approach had significant advantages in intraoperative bleeding and postoperative VAS pain scores compared with the unilateral intercostal approach. These results indicate that MSVATS can provide more convenient operation conditions, a better pleural cavity view, and a more complete thymectomy in the treatment of early thymoma, indicating that is a safe and feasible minimally invasive surgical method.

**Supplementary Information:**

The online version contains supplementary material available at 10.1186/s12885-024-11832-7.

## Background

Thymoma is a common anterior mediastinal tumor that arises from pathological changes in thymic lymph nodes or thymic epithelial cells and accounts for approximately 19–42% of mediastinal tumors [1, 2]. The majority of patients with thymoma are asymptomatic and not intentional at the time of diagnosis, while some patients present with chest tightness, chest pain, coughing, or difficulty breathing [3]. Postponement of treatment may cause associated complications such as aplastic anemia and myasthenia gravis (MG), and approximately 30–50% of patients with thymoma have myasthenia gravis and are diagnosed at the time of screening for MG, which can be life-threatening in severe cases [4, 5]. Therefore, it is important to diagnose and treat thymoma at an early stage. The primary treatment for patients diagnosed with thymoma is surgery, for which transthoracic thymectomy is the gold standard [6–8]. However, with the continual development of minimally invasive thoracoscopic equipment and techniques, thoracoscopic thymectomy is gradually being chosen by thoracic surgeons for its advantages of less intraoperative bleeding, less damage to surrounding tissues, less postoperative pain, and fewer complications.

Video thoracoscopic surgery (VATS) is broadly embraced as a minimally invasive thymectomy alternative to traditional median sternotomy for the treatment of early thymoma [9]. There are several minimally invasive thymectomy approaches available, including VATS thymectomy with a transcostal approach (IVATS), VATS thymectomy with a transcervical incision, and VATS thymectomy with a subxiphoid approach (SVATS) [9–11]. IVATS is the most widely adopted technique and remains commonly used. Currently, subxiphoid thoracoscopic thymectomy (SVATS) is gaining popularity among some thoracic surgeons. However, there is no high level of evidence to confirm the superiority of these two surgical approaches. Therefore, the purpose of this meta-analysis was to demonstrate the efficacy and safety of SVATS for the treatment of early-stage thymoma based on the available data.

Our previous study [12] described a modified subxiphoid approach to thoracoscopic thymectomy (MSVATS) for thymic carcinoma based on SVATS and progressively applied it to early thymoma. To investigate the clinical efficacy of modified subxiphoid approach thoracoscopic surgery for thymoma, we conducted a retrospective study of 117 patients who underwent thoracoscopic surgery for early thymoma at our hospital from September 2020 to March 2023.

## Materials and methods

### Study control

We conducted the search, data analysis, and writing independently. No other individuals were involved. The trial protocol can be found at PROSPERO under the registration number CRD42023407743.

### Search strategy and study selection

We conducted a comprehensive English language search using PubMed, Embase, and the Cochrane Library to find published articles on thoracoscopic surgery for early-stage thymoma reported up to March 1, 2023. During the data search, a combination of subject terms and free words was used, and references in the literature review section were reviewed retrospectively to supplement and obtain additional relevant information. Medical subject headings were used to search for terms such as thymoma, thoracoscopy, and subxiphoid. The reference lists of all full-text articles retrieved were screened to identify any additional potentially relevant studies.

### Selection criteria and data extraction

Publications meeting the following criteria were selected: (1) studies of thoracoscopic-assisted thymectomy; (2) randomized controlled trials (RCTs) or propensity-matched score studies; (3) comparison of unilateral versus subxiphoid access thymectomy; (4) early-stage thymoma (both stages I and II); and (5) reports including complete protocols, patient data, and at least one key clinical outcome, such as postoperative pain score, postoperative complication rate, operative time, time with tube, and postoperative hospital stay. Publications that met the following criteria were excluded: (1) the presence of inoperable or metastatic disease; (2) studies that did not focus on operative time, postoperative drainage, postoperative pain scores, or operative complication rates; (3) inclusion of fewer than 10 patients; (4) robotic thymectomy or median sternal split thymectomy; (5) duplicate publications; and (6) violation of any of the above inclusion criteria. Two investigators reviewed all studies in the literature retrieved from this independent search and excluded any irrelevant studies by scanning titles and abstracts. The studies in the literature that met our meta-analysis were then screened by reading their full texts after downloading them according to the inclusion and exclusion criteria mentioned above. In case of disagreement between the two investigators, the decision was made by a third investigator after a careful reading of the full text.

### Data extraction

Two investigators carefully read each report in its entirety and independently collected data, and a senior statistician confirmed the study investigators’ data. Outcome indicators collected included some or all of the data described below, such as BMI, tumor size, length of surgery, intraoperative bleeding, postoperative drainage time, postoperative drainage, postoperative hospital days, visual analog score (VAS) scores on days 1, 3, and 7 of surgery, and postoperative complications. For missing data, we sought assistance from the authors of the original studies whenever possible.

### Surgical approach

#### Modified subxiphoid process

**Fig. 1:**
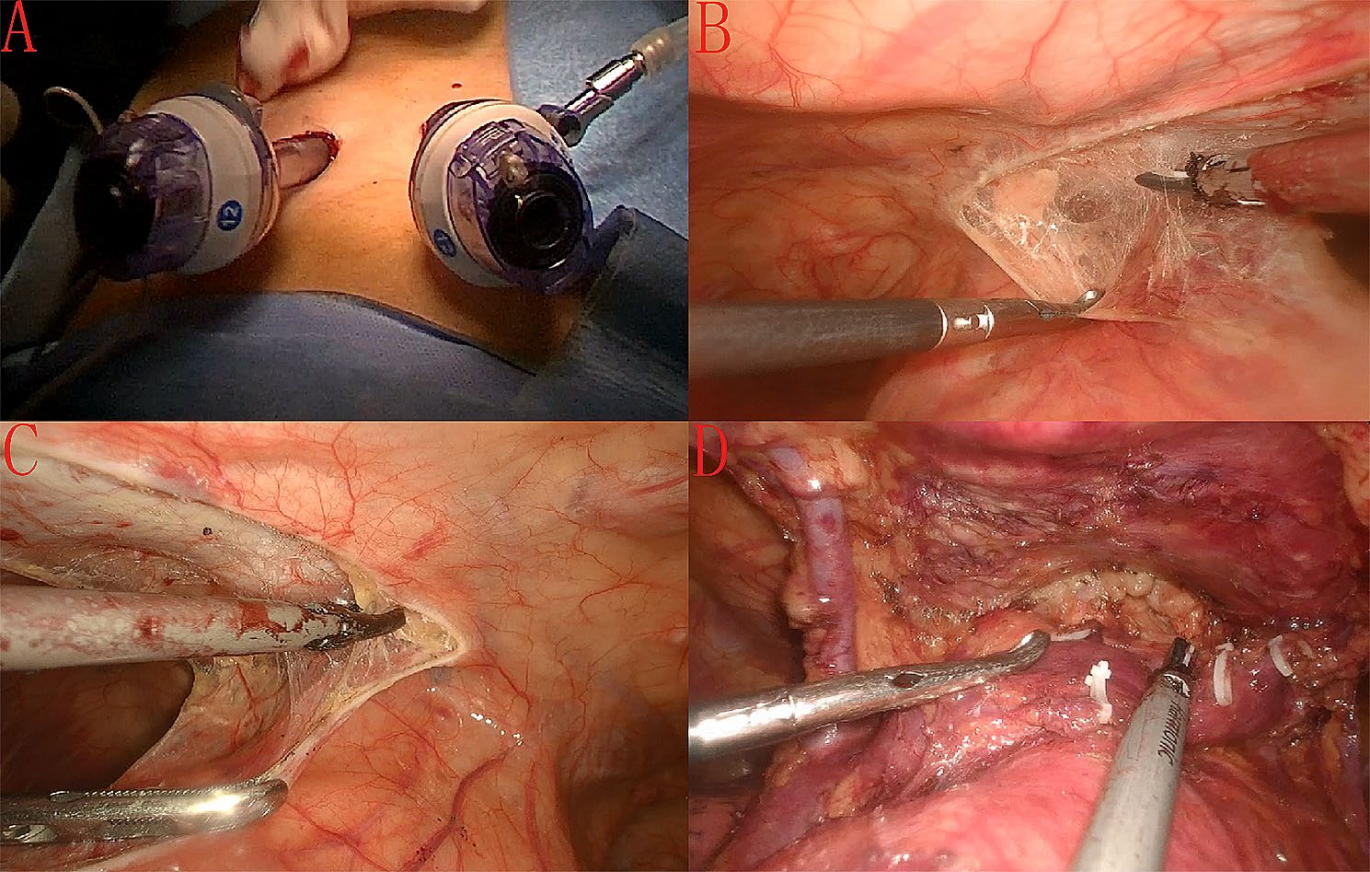
Intraoperative images: (**A**) incision location; (**B**) right side view; (**C**) left side view; (**D**) exposure of the left innominate vein

Based on our previous study [12]: The patient was placed on the operating table in the supine position, and the legs were spread apart, with the operator on the right side of the patient and the assistant in the middle of the legs spread apart. A single-lumen endotracheal tube was inserted under general anesthesia with bilateral pulmonary low-flow ventilation. A 1.5-cm incision was made bilaterally under the costal arches 3 cm below the xiphoid process. The rectus abdominis muscle was incised, and the fingers entered the incision bilaterally and bluntly separated the posterior sternal space before converging and placing separate Conkey 12 poke cards. A thoracoscope was placed in the left costal arch incision, and 8–12 cm H_2_O positive-pressure carbon dioxide (CO_2_) was injected to induce mediastinal emphysema, which loosened the connective tissue and widened the retrosternal space. An ultrasonic knife was placed in the right costal arch incision to open the bilateral mediastinal pleura. A 0.5-cm incision was made at the junction of the right sixth rib and the anterior axillary line, and a Conkey 5 poke card and grasping forceps were placed. The lower pole of the thymus was first lifted with the grasping forceps to expose the ascending aorta and the innominate vein. The fat pad near the phrenic nerve was also incised with the ultrasonic knife. Careful dissection of the cardiophrenic angle, aortopulmonary window, prepericardial fat, and fat pads near the inferior end of the thyroid gland was performed. The resection area included the upper and lower poles of the thymus and the thymic tumor, including the mediastinal pleura and the pericardial adipose tissue within the phrenic nerve bilaterally. The superior vena cava, innominate vein, and aorta were ossified. All specimens were placed in a specimen bag and removed from the right costal arch incision. An incision was made at the junction of the right sixth rib and the anterior axillary line, a drain was inserted obliquely upward to the mediastinum, and the skin incision was closed (Fig. 1).

#### Lateral thoracic approach

The patient was placed under general anesthesia, changed from single-lumen tracheal intubation to double-lumen intubation, and ventilated unilaterally with one lung. Surgical position: The patient was placed in a 30–45° semi-supine position. A 1.5-cm incision between the sixth intercostal space in the right mid-axillary line was generally chosen as the observation hole for the insertion of a trocar needle. A 3-cm incision was made at the fourth intercostal space on the right side of the posterior axillary line as the primary surgical hole, with the position of the incision adjusted according to the requirements of the operation. A 1.5-cm incision was made at the sternum, and a trocar needle was used as a secondary surgical hole. Then, an ultrasonic knife was used to open the mediastinal pleura along the dorsal aspect of the intrathoracic artery, the superior vena cava, and the anterior aspect of the right phrenic nerve. The area was then carefully analyzed, the free fat of the anterior mediastinum and the right lobe of the thymus were exposed, the thymus was visibly separated at the right lower pole at the base of the heart, the right lobe of the thymus was pulled back to the right, and the left lobe of the thymus was separated from the left lower pole. The thymus was lifted upward, the thymic vein was isolated at the angle of venous entrapment, and the titanium clamp was closed and severed to completely separate the right upper pole of the thymus. The thymus tissue was removed, the left upper pole was separated, and the thymus tissue was completely.

#### Patients

The patients and their families were informed of the advantages and risks of this new approach prior to surgery, and informed consent was obtained for a possible switch to the sternal approach if there was significant vascular injury or if a new surgical approach was not an option. In addition, each patient and his or her family provided written informed consent, which was approved by the Institutional Review Committee or Ethics Committee of the Second Hospital of Jilin University. At our hospital, a team of experienced neurologists and thoracic surgeons work together to treat patients diagnosed with thymoma. A retrospective collection of clinical data from all Masaoka stage I and II thymoma patients who underwent subxiphoid and lateral thoracoscopic thymectomy from September 2020 to March 2023 was performed. The initial diagnosis of thymoma was based on computed tomography (CT) imaging of the chest and the clinical presentation. Staging of each thymoma was evaluated according to Masaoka’s staging system [13]. The inclusion criteria were as follows: (1) preoperative confirmation of an anterior mediastinal mass with a lesion diameter ≤ 5 cm and no vascular or peripheral tissue invasion; and (2) stage I–II with Masaoka staging and no metastases. The exclusion criteria were as follows: (1) patients who were transferred to an open approach intraoperatively; (2) patients with severe MG; (3) patients with incomplete palliative surgical resection due to intraoperative bleeding or long thymic diameter > 5 cm; and (4) imaging indications of preoperative vascular or peripheral tissue invasion or distant metastasis based on postoperative pathology.

### Statistical analysis

Review Manager version 5.4 (RevMan; Cochrane Collaboration), a professional software program provided by the Cochrane Collaboration, was used [14]. For statistical analysis, relative ratios (RRs) were used for binary variables, and mean differences (MDs) or normalized mean differences (SMDs) were used for continuous variables with 95% confidence intervals (CIs). The χ^2^ and I^2^ tests were used to determine heterogeneity. To determine that the joint results were not heavily influenced by individual tests, the included studies were removed sequentially for sensitivity analysis. A random-effects model was used when heterogeneity was significant; additionally, a fixed-effects model was used. *P* < 0.05 was considered a statistically significant difference. A Higgins I^2^ statistic < 50% was considered low heterogeneity, and > 50% was considered high heterogeneity. Subgroup analyses were performed to identify sources of heterogeneity and factors associated with clinical outcomes. SPSS 28.0 software was used for statistical analysis. Continuous variables conforming to a normal distribution were expressed as means ± standard deviations and the t-test was used. Categorical variables were expressed as the number of cases (%), and the chi-square test or Fisher’s exact probability method was used. The rank-sum test was performed for ordered variables and continuous variables that did not conform to a normal distribution.

### Assessments of publication bias and study quality

The quality of the included studies was assessed using the recommended risk of bias assessment tools provided in the Cochrane Handbook 5.1.0, including (1) random assignment method; (2) allocation concealment; (3) whether participants and researchers were blinded; (4) whether results were assessed using blinded methods; (5) completeness of the outcome data; (6) selective reporting of results; and (7) other biases. Qualitative evaluations were performed independently by two investigators, and disagreements were discussed by the two investigators or resolved by a third investigator. Possible publication bias in clinical studies was examined using funnel plots.

## Results

### Results of the literature search

**Fig. 2:**
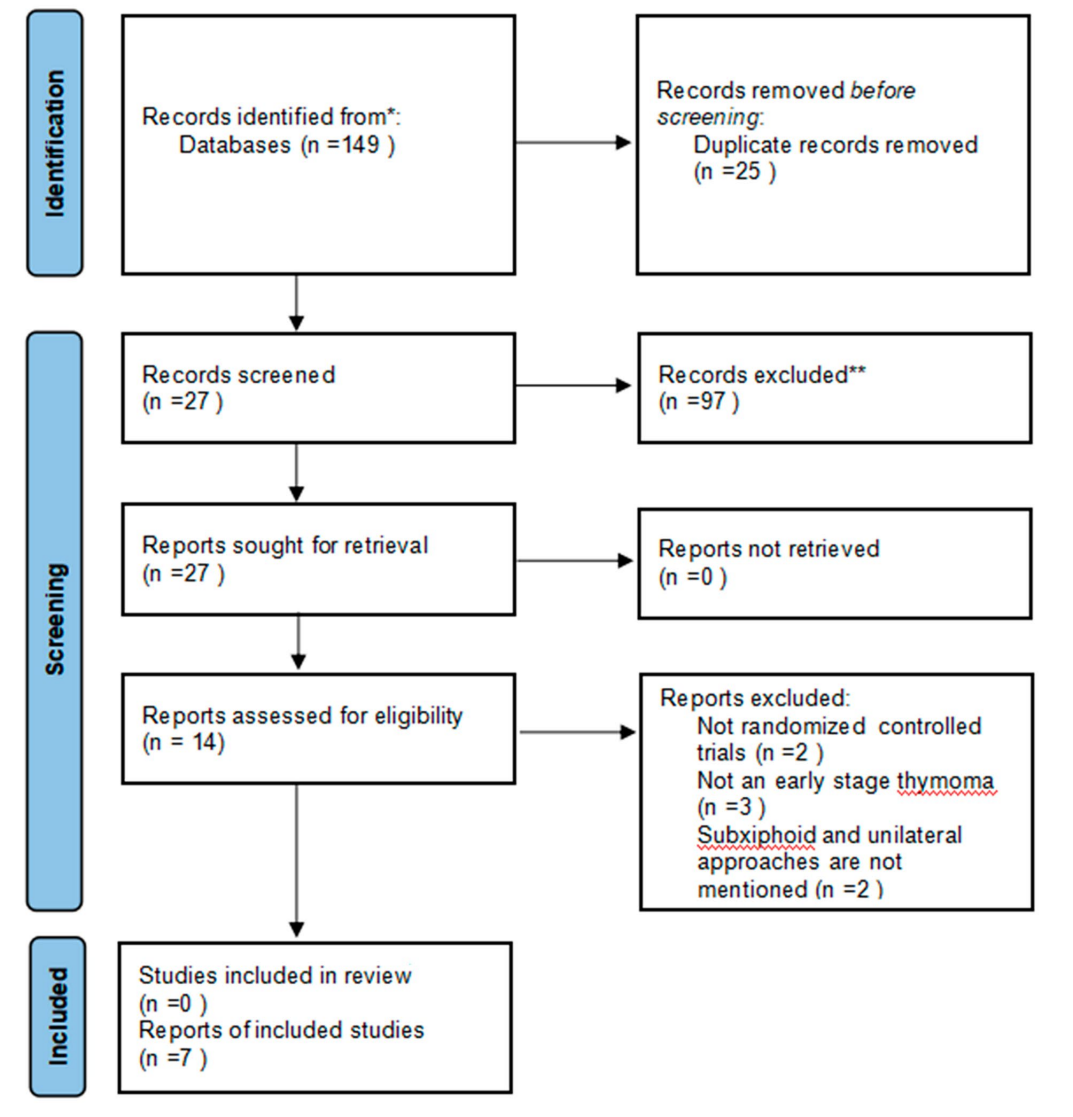
Publication search

**Fig. 3:**
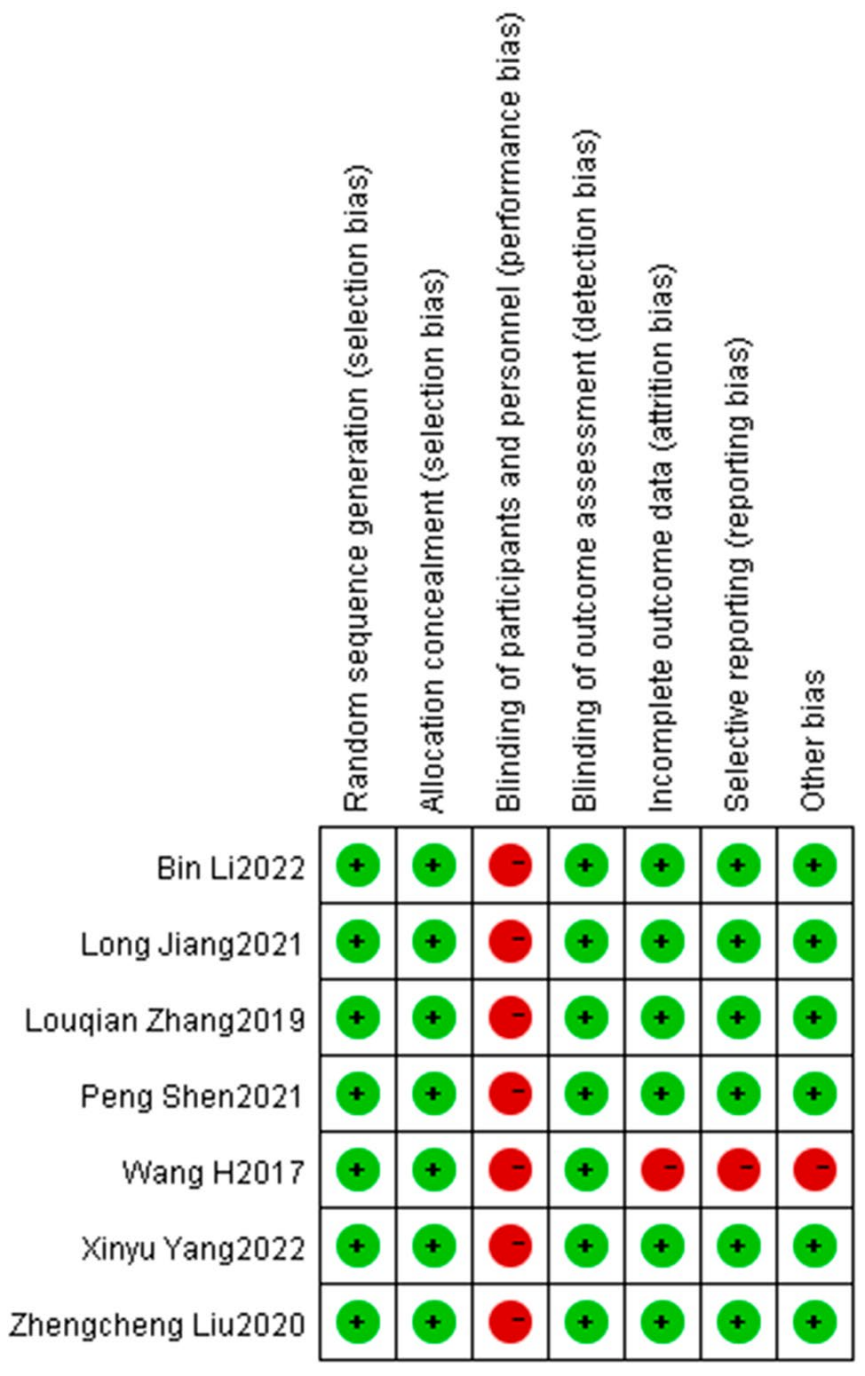
Assessments of publication bias and study quality

In accordance with the study strategy, 149 documents were retrieved in the first search, 25 duplicates were removed, 97 were removed based on title and abstract, and 27 were ultimately selected for a full detailed examination. A total of 14 full-text articles were available free of charge, and after careful reading of the full texts, seven studies were excluded because they did not meet the inclusion criteria. A total of 1607 patients were included in the seven studies [4, 15–20] and were used for the analyses. Of these, 591 patients underwent thoracoscopic-assisted subxiphoid approach thymectomy, and 1016 patients underwent thoracoscopic-assisted lateral thoracic approach thymectomy. The detailed study selection process is shown in Fig. 2, and the low risk of summary bias for the included studies is summarized in Fig. 3. Further details of the patient characteristics in the meta-analysis are shown in Tables 1, 2 and 3.

**Table 1:** Basic characteristics of patients included in the study(SVATS/IVATS)

Study Atuhor	Year	Country	Stage	Number of patients	Age	BMI	Size
Zhengcheng Liu	2020	China	I, II	76/217	49.8 ± 9.2/51.3 ± 10.2	23.1 ± 2.9/23.9 ± 3.2	3.2 ± 1.8/3.5 ± 2.1
Peng Shen	2021	China	I, II	45/45	65.21 ± 1.23/65.23 ± 1.22	25.37 ± 1.59/25.42 ± 1.61	4.05 ± 1.41/ 4.11 ± 1.35
Bin Li	2022	China	I, II	99/246	49.05 ± 12.56/50.69 ± 13.89	24.35 ± 3.48/24.26 ± 3.90	3.74 ± 0.76/3.72 ± 1.09
Xinyu Yang	2022	China	I, II	268/193	54.1 ± 12.6/56.2 ± 11.7	22.8 ± 2.4 /23.1 ± 2.3	4.9 ± 2.3/4.9 ± 2.7
Louqian Zhang	2019	China	I, II	28/70	58.2 ± 10.0/54.8 ± 8.6	22.7 ± 3.8/ 23.4 ± 2.6	3.2 ± 1.6/3.6 ± 1.3
Long Jiang	2021	China	I,II	39/198	48 ± 14/47 ± 14	23.2 ± 3.1/23.1 ± 3.3	6.7 ± 2.9/5.9 ± 2.8
Wang H	2017	China	Early stage	36/47	-	-	-

**Table 2:** Basic characteristics of patients included in the study(SVATS/IVATS)

Study Atuhor	Year	Operation time	Intraoperative blood loss	Diversion flow	Tube time	Postoperative hospital stay
Zhengcheng Liu	2020	88 ± 29/81 ± 41	55 ± 40/46 ± 35	-	2.2 ± 1.1/2.5 ± 1.3	-
Peng Shen	2021	89.24 ± 10.53/145.25 ± 13.75	46.21 ± 8.98/59.47 ± 11.12	171.22 ± 43.28/231.82 ± 44.37	-	3.25 ± 1.11/5.29 ± 1.38
Bin Li	2022	97.83 ± 29.05/98.71 ± 42.47	32.02 ± 41.48/56.87 ± 82.01	240.03 ± 169.54/359.01 ± 461.74	2.77 ± 0.82/3.53 ± 1.94	3.75 ± 1.02/4.40 ± 2.09
Xinyu Yang	2022	72.13 ± 18.06/64.46 ± 15.77	8.69 ± 8.50/9.74 ± 5.36	205.59 ± 82.03/215.07 ± 84.43	2.38 ± 0.49/2.31 ± 0.51	2.99 ± 0.82/2.78 ± 0.95
Louqian Zhang	2019	104 ± 29/116 ± 36	40(30–70)/40 (30–80)	-	1.8 ± 1.6/2.1 ± 1.3	3.6 ± 1.2/4.3 ± 1.6
Long Jiang	2021	147.5 ± 43.6/103.5 ± 58.7	35.8 ± 42.4/65 ± 211.7	341.3 ± 411.1/379.8 ± 377.2	1.8 ± 1.5 /1.5 ± 1.7	4.2 ± 2.1/3.9 ± 2.6
Wang H	2017	63.5 ± 10.7/87.7 ± 13.1	-	-	1.6 ± 0.6/2.3 ± 0.9	-

**Table 3:** Basic characteristics of patients included in the study(SVATS/IVATS)

Study Atuhor	Year	Postoperative complication	Postoperative pain score (VAS) Day 1	Postoperative pain score (VAS) Day 3	Postoperative pain score (VAS) Day 7
Zhengcheng Liu	2020	-	1.5 ± 0.4/3.3 ± 1.2	1.2 ± 0.3/2.9 ± 0.7	1.1 ± 0.3/1.9 ± 0.5
Peng Shen	2021	1/45 / 6/45	-	-	-
Bin Li	2022	11/99 / 18/246	3.12 ± 0.70/4.59 ± 0.79	-	-
Xinyu Yang	2022	6/144 / 10/144	2.67 ± 1.45/4.24 ± 1.53	2.07 ± 0.84/2.86 ± 0.64	0.80 ± 0.28/ 1.01 ± 0.40
Louqian Zhang	2019	0/28 / 5/65	-	-	-
Long Jiang	2021	10/39 / 22/198	2.9 ± 1/5.8 ± 1.3	2.2 ± 0.6/4 ± 1	-
Wang H	2017	2/36 / 3 /47	-	-	-

### Primary outcomes of the search

**Fig. 4:**
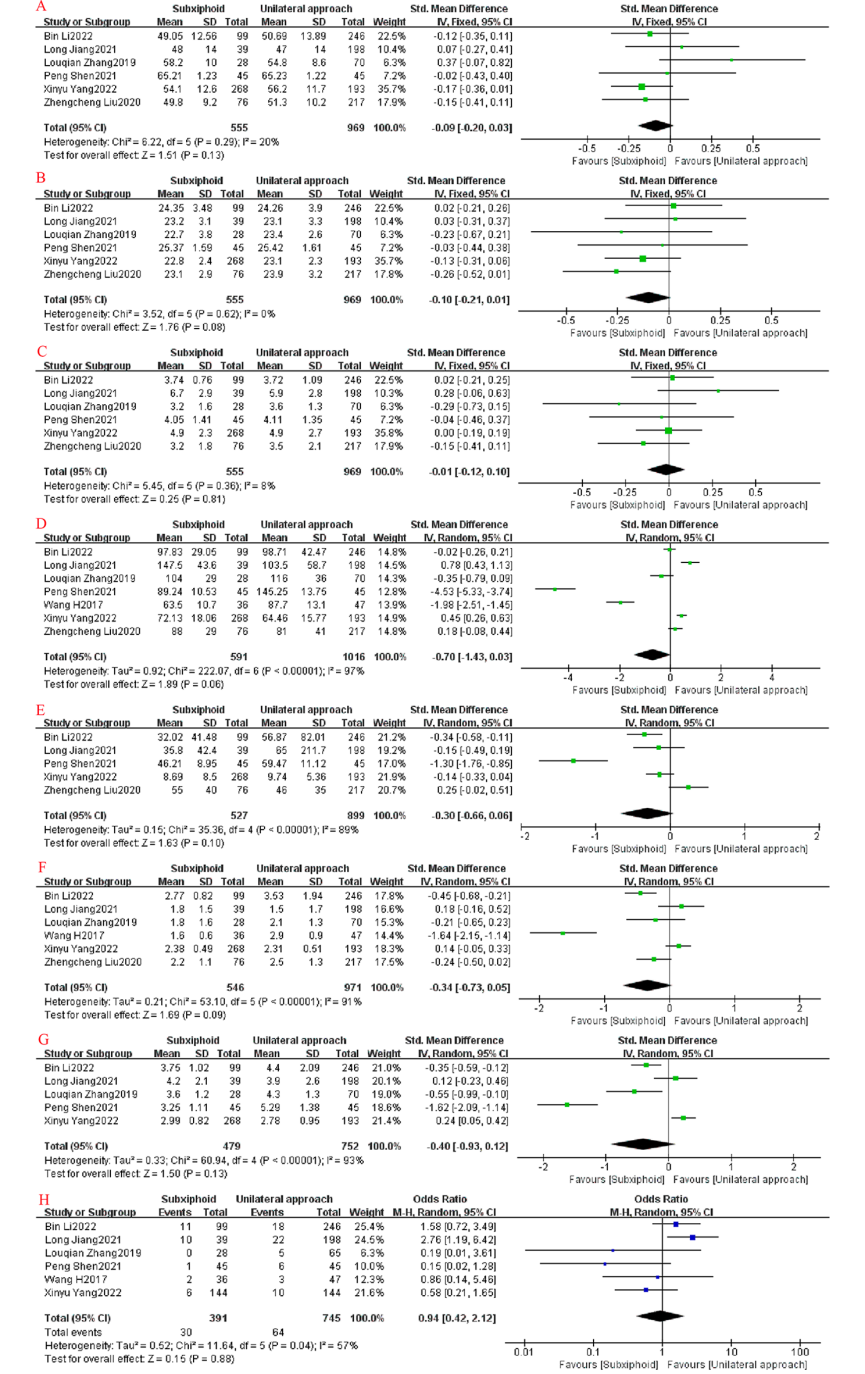
Forest plots: (**A**) age; (**B**) BMI; (**C**) thymoma size; (**D**) operative time; (**E**) intraoperative bleeding; (**F**) time to extubation; (**G**) postoperative hospital stay; (**H**) postoperative complications

In all the included studies, we found no statistically significant differences in age (SMD = − 0.09, 95%CI: −0.20 to − 0.03, I^2^ = 20%, *p* = 0.13), body mass index (BMI; SMD = − 0.10, 95%CI: −0.21 to − 0.01, I^2^ = 0%, *p* = 0.08), thymoma size (SMD = − 0.01, 95%CI: −0.12–0.10, I^2^ = 8%, *p* = 0.81), operative time (SMD = − 0.70, 95%CI: −1.43–0.03, I^2^ = 97%, *p* = 0.06), intraoperative bleeding (SMD = − 0.30, 95%CI: −0.66–0.06, I^2^ = 89%, *p* = 0.10), time to extubation (SMD = − 0.34, 95%CI: −0.73–0.05, I^2^ = 91%, *p* = 0.09), postoperative hospital stay (SMD = − 0.40, 95%CI: −0.93–0.12, I^2^ = 93%, *p* = 0.13), and postoperative complications (odds ratio [OR] = 0.94, 95%CI: 0.42–2.12, I^2^ = 57%, *p* = 0.88) between the SVATS and IVATS groups (Fig. 4).

**Fig. 5:**
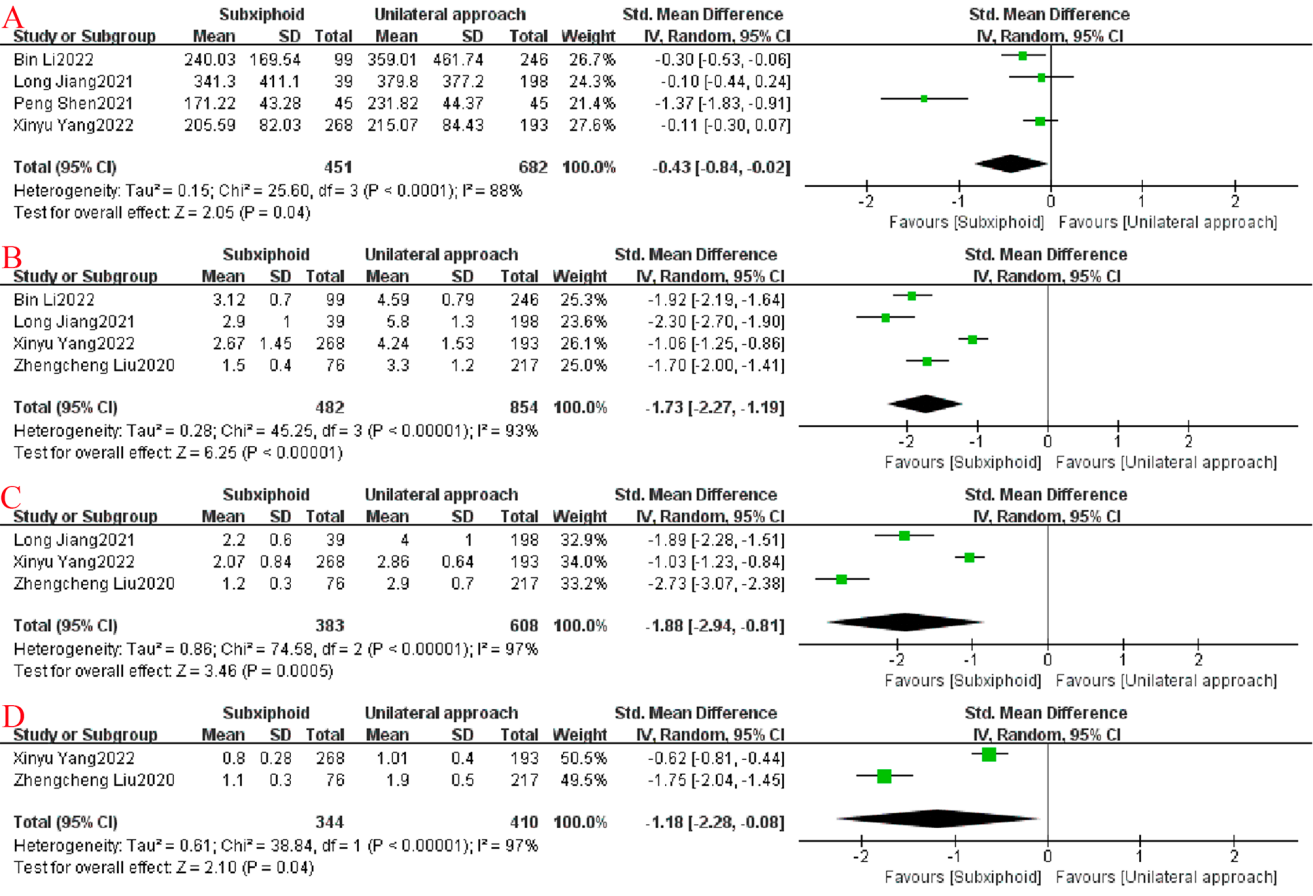
Forest plots: (**A**) postoperative drainage; (**B**) postoperative pain score (VAS) day 1; (**C**) postoperative VAS day 3; (**D**) postoperative VAS day 7

In addition, the postoperative drainage in the SVATS group was less than that in the IVATS group (SMD = − 0.43, 95%CI: −0.84 to − 0.02, I^2^ = 88%, *p* = 0.04), and the difference was statistically significant. More importantly, the postoperative VAS was lower in the SVATS group on days 1 (SMD = − 1.73, 95%CI: −2.27 to − 1.19, I^2^ = 93%, *p* < 0.00001), 3 (SMD = − 1.88, 95%CI: −2.84 to − 0.81, I^2^ = 97%, *p* = 0.0005), and 7 (SMD = − 1.18, 95%CI: −2.28 to − 0.08, I^2^ = 97%, *p* = 0.04) than in the IVATS group, and these differences were statistically significant (Fig. 5).

### Sensitivity analysis

**Fig. 6:**
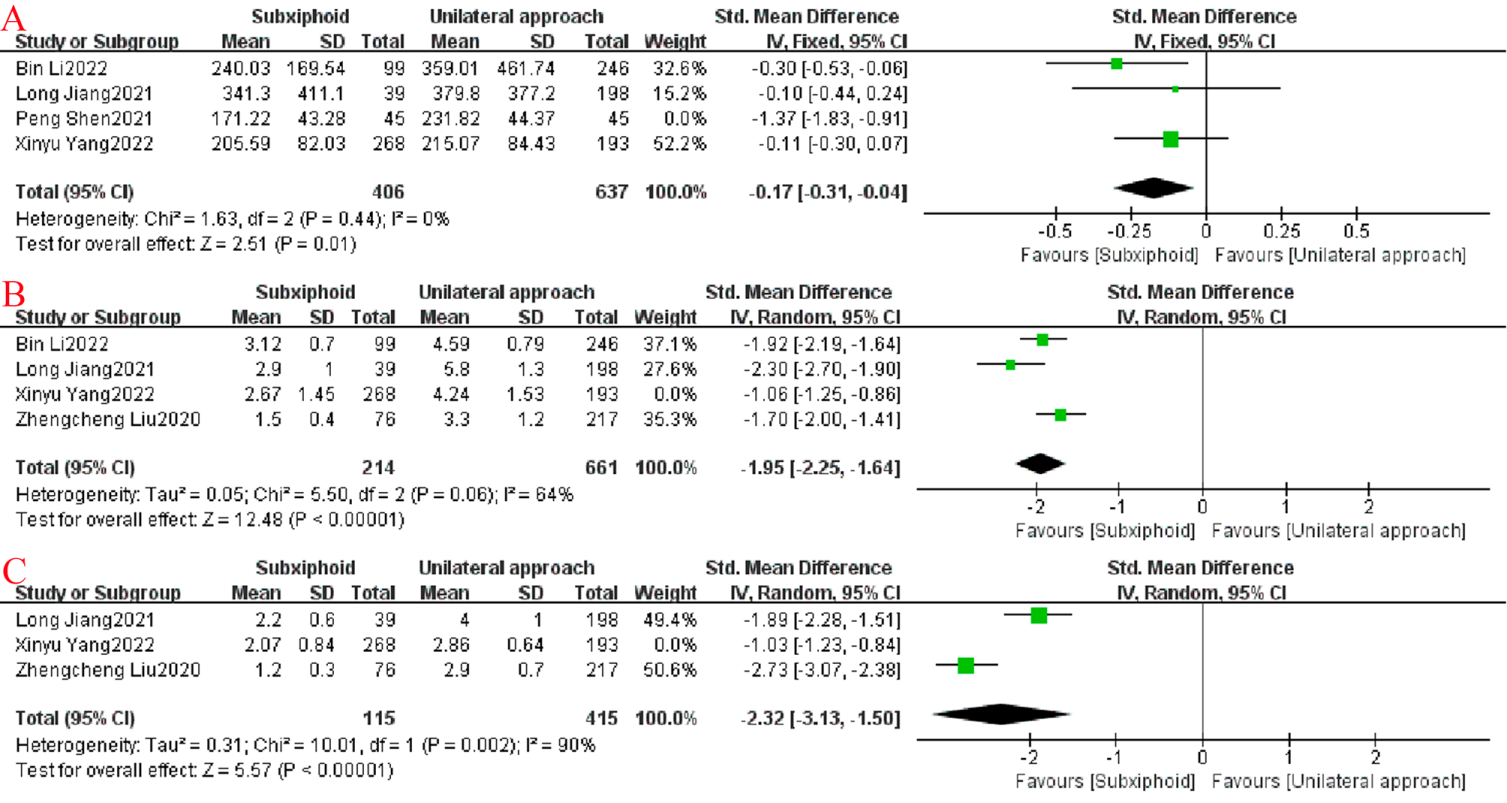
Sensitivity analysis forest plots: (**A**) postoperative drainage; (**B**) postoperative pain score (VAS) day 1; (**C**) postoperative VAS day 3

A reinspection of study search, selection, and inclusion criteria did not reduce the heterogeneity. To determine that joint results were not heavily influenced by individual trials, a sensitivity analysis of included studies was performed. In a single-study analysis of postoperative drainage in four studies, a study by Peng Shen et al. [4] was the most important factor for heterogeneity but did not have the greatest weight among the studies. Excluding the significant reduction in heterogeneity after removing this study (*p* = 0.01; I^2^ = 0%), the combined results of the remaining three trials still significantly demonstrate that postoperative drainage was less in the SVATS group than in the IVATS group. By contrast, in exploring the heterogeneity of VAS postoperative day 1 scores (*p* < 0.00001; I^2^ = 64%) and VAS postoperative day 3 scores (*p* < 0.00001; I^2^ = 90%), the removal-by-removal study revealed that although the heterogeneity was not significantly lower in the SVATS group compared with the IVATS group, the difference was still statistically significant (Fig. 6).

### Publication bias test

**Fig. 7:**
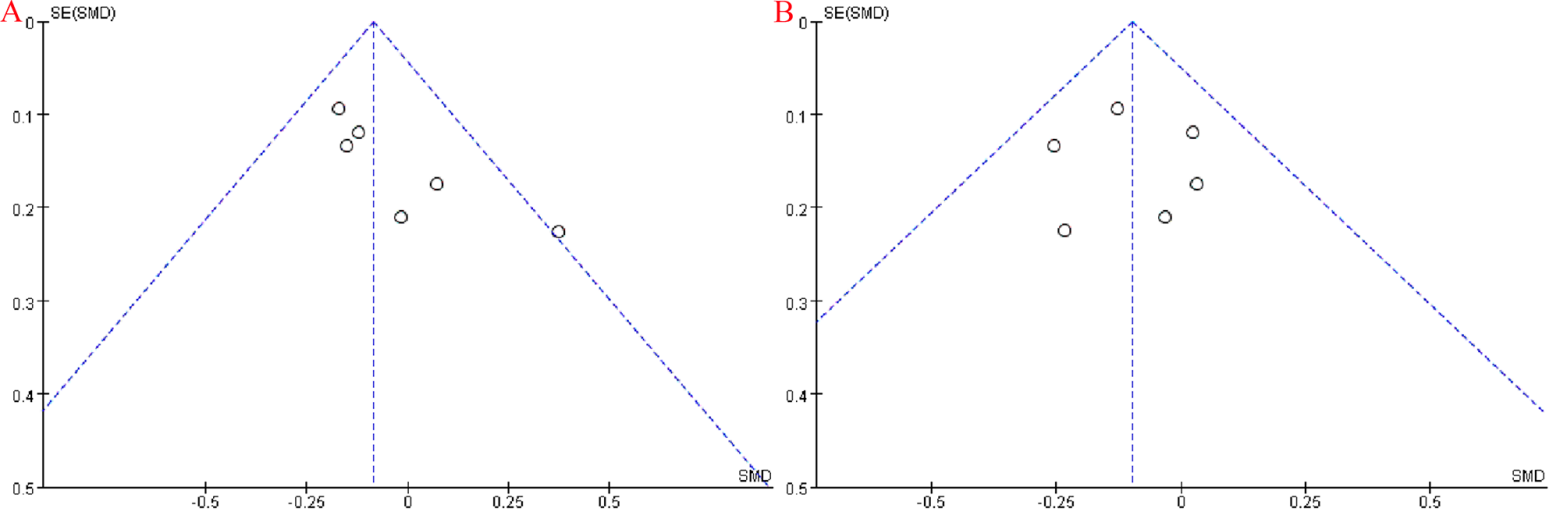
Publication bias tests: (**A**) age; (**B**) BMI

In the analysis of studies in the SVATS and IVATS groups for early-stage thymoma, funnel plots were used to examine possible publication bias among the seven clinical studies. The images revealed a symmetrical funnel plot distribution with no significant publication bias (Fig. 7).

### Patient characteristics

The characteristics of the 117 patients with early anterior mediastinal thymoma in the Department of Thoracic Surgery, Second Affiliated Hospital of Jilin University from September 2020 to March 2023 were retrospectively collected. After surgery, all patients were clinically staged between stages I and II according to Masaoka staging, which included 42 patients who received MSVATS and 75 patients who received IVATS. For the MSVATS group, among the 42 patients, 25 were male and 17 were female, their age was 43.64 ± 11.54 years, their BMI was 23.48 ± 3.00 kg/m^2^, their tumor size was 3.04 ± 0.73 cm, and the incidence of preoperative muscle weakness was 11.9% (5/42). For the IVATS group, among the 75 patients, 39 were male and 36 were female, their age was 44.57 ± 11.12 years, their BMI was 23.71 ± 2.21 kg/m^2^, their tumor size was 2.91 ± 0.71 cm, and the incidence of preoperative muscle weakness was 9.3% (7/75). All IVATS groups underwent complete thymectomy, while the MSVATS groups underwent extended thymectomy.

The differences in general clinical characteristics, including age, sex, tumor diameter, Masaoka stage, and World Health Organization (WHO) stage, between the two groups were not statistically significant (*p* > 0.05). However, the MSVATS group had a slightly smaller BMI than the IVATS group, and the difference was statistically significant (*p* < 0.05) (Table 4).

**Table 4:** Comparison of the clinical characteristics between the two groups

	MSVATS(*n* = 42)	IVATS(*n* = 75)	*P*-value
Age	43.64 ± 11.54	44.57 ± 11.12	0.768
Gender			0.433
Male	25	39	
Female	17	36	
BMI (kg/m2)	23.48 ± 3.00	23.71 ± 2.21	0.005
Tumor size(cm)	3.04 ± 0.73	2.91 ± 0.71	0.416
Masaoka stage			0.578
Phase I	38	70	
Phase II	4	5	
WHO histologic type			0.907
A/AB	29	51	
B1/B2/B3	13	24	
Preoperative myasthenia	5/42	7/75	0.660

The intraoperative and postoperative conditions in the MSVATS and IVATS groups are shown in Table 5. The MSVATS group had lower values for operative time (78.26 ± 14.61 vs. 91.76 ± 10.84, *p* = 0.233), postoperative drainage (193.10 ± 40.03 vs. 273.47 ± 34.50, *p* = 0.475), extubation time (2.83 ± 0.91 vs. 3.39 ± 0.96, *p* = 0.744), postoperative hospital stay (3.52 ± 0.89 vs. 4.15 ± 0.94, *p* = 0.807), and postoperative complication rate (0/42 vs. 6/75, *p* = 0.060) than those in the IVATs group, but the differences were not statistically significant.

**Table 5:** Comparison of intraoperative and postoperative indicators between the two groups

	MSVATS(*n* = 42)	IVATS(*n* = 75)	*P*-value
Operation time(min)	78.26 ± 14.61	91.76 ± 10.84	0.233
Intraoperative blood loss(ml)	37.38 ± 11.27	52.47 ± 5.72	0.000002
Postoperative drainage(ml)	193.10 ± 40.03	273.47 ± 34.50	0.475
Tube time(day)	2.83 ± 0.91	3.39 ± 0.96	0.744
Postoperative hospital stay(day)	3.52 ± 0.89	4.15 ± 0.94	0.807
Postoperative complication	0/42	6/75	0.060
Postoperative pain score (VAS) Day 1	1.86 ± 0.81	3.61 ± 1.10	0.001
Postoperative pain score (VAS) Day 3	1.45 ± 0.77	2.65 ± 0.98	0.045
Postoperative pain score (VAS) Day 7	0.93 ± 0.51	1.25 ± 0.70	0.0001

However, the MSVATS group also had lower values for intraoperative bleeding (37.38 ± 11.27 vs. 52.47 ± 5.72, *p* = 0.00002), VAS on postoperative day 1 (1.86 ± 0.81 vs. 3.61 ± 1.10, *p* = 0.001), VAS on postoperative day 3 (1.45 ± 0.77 vs. 2.65 ± 0.98, *p* = 0.045), and VAS on postoperative day 7 (0.93 ± 0.51 vs. 1.25 ± 0.70, *p* = 0.0001) than those in the IVATS group, and the differences were statistically significant.

## Discussion

The current treatment modalities for thymoma are mainly surgical, with surgical thoroughness remaining a key factor in patient survival [21, 22]. According to the International Thymic Malignancy Interest Group, thymectomy should involve the complete removal of the tumor, thymus, and anterior mediastinal adipose tissue [23]. Transthoracic thymectomy has been the gold standard for the surgical treatment of thymoma since Blalock proposed it in 1939 [8, 24]. Because of the continuous development of endoscopic technology, it has become the main method for performing thymectomies. Based on the National Comprehensive Cancer Network (NCCN) guidelines, thoracoscopic thymectomy is indicated for the treatment of Masaoka–Koga stages I and II thymomas without MG [25]. Thoracoscopic thymectomy along the intercostal space is currently the most commonly used surgical approach, but the transcostal approach makes it difficult to expose the contralateral phrenic nerve and anterior mediastinum, which may result in residual thymic tissue being left after surgery, introducing a potential risk for future recurrence [11, 26]. Moreover, the superior pole of the thymus is inconveniently exposed via the intercostal space, which may result in cephalothoracic vein injury [11, 27]. Therefore, some thoracic surgeons opt for bilateral intercostal thoracoscopic thymectomy. Kido et al. first reported SVATS thymectomy in 1999 and noted that this surgical method could solve the above problems [23]. Several studies have confirmed the safety of the subxiphoid approach in the treatment of anterior mediastinal lesions [28–30]. The SVATS allows for adequate visualization of the entire anterior mediastinal space during surgery for early-stage thymomas with tumor sizes < 5 cm. With this approach, expanded thymectomy and adipose tissue stripping are allowed. With the identification of the right and left phrenic nerves, according to the ITMIG guidelines, the anterior mediastinal adipose tissue must be removed to ensure radicalization of the tumor and nerves, followed by dissection from the jugular vein to the anterior pericardial diaphragm angle [31]. Since then, the subxiphoid approach has gradually become the surgical approach of choice for thoracic surgeons.

This meta-analysis revealed no significant differences in operative time, intraoperative bleeding, extubation time, postoperative hospital stay, or postoperative complication rates between the SVATS and IVATS groups. However, the SVATS group had significantly reduced postoperative drainage and postoperative VAS scores. We consider that these differences may be related to the fact that the IVATS approach causes varying degrees of damage to the intercostal nerves, whether unilateral or bilateral, leading to long-term postoperative chest pain and numbness [32].

The advantages and disadvantages of both MSVATS and IVATS approaches were also compared in our meta-analysis. The differences between the two groups (MSVATS vs. IVATS) in general clinical characteristics such as age, sex, tumor diameter, Masaoka stage, WHO stage, and intraoperative and postoperative conditions, including operative time, postoperative drainage, extubation time, postoperative hospital stay, and postoperative complication rates were not statistically significant (*p* > 0.05), while the MSVATS group had lower values for BMI, intraoperative bleeding, and VAS on postoperative days 1, 3, and 7 that were all statistically significant (*p* < 0.05) compared with those in the IVATS group. Thus, the modified subxiphoid approach had a significant advantage in reducing intraoperative blood loss but was no better than the conventional subxiphoid approach in terms of postoperative VAS scores. No perioperative deaths were recorded among any of the included patients. No patients in the MSVATS/IVATS groups were referred to sternal approaches for reasons such as intraoperative bleeding. By contrast, in the study by Long Jiang et al [19], three and two patients required transfer to the sternal approach in the subxiphoid and unilateral intercostal approaches, respectively. The reasons for transfer were: tumor penetration of the pericardium (3), invasion of the pericardium and the recurrent laryngeal nerve (1), and large pleural adhesions (1). Moreover, in the study by Wang H et al., one patient was referred to the sternal approach due to bleeding [20]. No postoperative complications occurred in the MSVATS group, while the IVATS group had an 8% (6/75) postoperative complication rate, which included pneumonia, delayed wound healing, and cardiac arrhythmias, as described in the study by Bin Li and colleagues [16]. Postoperative myasthenia crisis after thymectomy has been reported to occur at rates ranging from 6–34% [33]. However, postoperative myasthenia crisis was not mentioned in this meta-analysis and did not occur in this study. In our study, we found that patients with high BMI (obesity) or abdominal obesity did not benefit from MSVATS, mainly due to the narrowing of the chest cavity caused by the increased difficulty of blunt finger separation of subxiphoid tissues due to excess fat or muscle, as well as diaphragmatic rise.

Thoracic surgeons also have new surgical options when performing thymectomies with the development of da Vinci Robot-Assisted Thoracic Surgery (RATS) [34]. The advantage of RATS is that it provides the operator with 10x magnification and the physical conditions for 3D image effects. It also has a very flexible operating arm that can move in seven dimensions. During surgery, the surgeon can perform the procedure while seated, reducing physical exertion and elevating concentration. This technology offers great advantages to thoracic surgeons, especially for the removal of lesions located in narrow spaces and in areas where important neurovascular structures are located, such as the mediastinum [35, 36]. A total of 11 studies comprising 1418 patients were included in a meta-analysis of VATS versus RATS by Cheng Shen et al [37], with 688 patients included in the RATS group and 730 patients in the VATS group. Compared with VATS, RATS was associated with less intraoperative bleeding, less drainage, fewer days of postoperative chest drainage, shorter postoperative hospitalization, and fewer postoperative complications. There were no significant differences between the two groups in terms of operative time and the presence or absence of patients with MG. The advantages of RATS are more obvious, but because of economic constraints, this modality cannot be popularized to all levels of hospitals, while thoracoscopy is more readily available.

In contrast to the ideas previously suggested by investigators, it was possible to adequately expose the anterior mediastinum, bilateral phrenic nerves, left innominate vein, right internal mammary vein, and bilateral pericardial fat pads by MSVATS, which enabled more patients who could not tolerate single-lung ventilation to undergo the procedure and facilitated operator manipulation with as little interference as possible with assistant instruments. Altering the entrance of the thoracoscope into the bilateral costal arches and constricting it allows better exposure of the supradiaphragmatic adipose tissue and thymic tissue around the main pulmonary artery window to facilitate extended thymectomy. Of course, there are still some limitations to this surgical approach. First, the diameter of the thymic tumor may be too large to be removed from a minimally invasive incision. Secondly, subxiphoid surgery is not suitable for patients with large hearts in whom exposure of the anterior mediastinum is incomplete. Thus, there is still a need for large randomized controlled clinical trials to demonstrate the safety and efficacy of this procedure.

In summary, this meta-analysis revealed that the conventional subxiphoid approach was superior in terms of postoperative drainage and postoperative VAS pain scores compared with the unilateral intercostal approach. The modified subxiphoid approach also had significant advantages in intraoperative bleeding and postoperative VAS pain scores compared with the unilateral intercostal approach. MSVATS can provide more convenient operation conditions, a better pleural cavity view, and a more complete thymectomy in the treatment of early thymoma, indicating that is a safe and feasible minimally invasive surgical method.

### Electronic supplementary material

Below is the link to the electronic supplementary material.

Supplementary Material 1

## Data Availability

The authors confirm that data supporting the results of this study are available in the paper. Further enquiries may be directed to the corresponding author.
